# Impact of Storage Conditions on EV Integrity/Surface Markers and Cargos

**DOI:** 10.3390/life12050697

**Published:** 2022-05-07

**Authors:** Ayyanar Sivanantham, Yang Jin

**Affiliations:** Division of Pulmonary and Critical Care Medicine, Department of Medicine, Boston University School of Medicine, Boston, MA 02118, USA; ayyanars@bu.edu

**Keywords:** extracellular vesicles (EVs), biotherapeutics, long-term storage, stability, temperature, freeze-thaw cycle

## Abstract

Extracellular vesicles (EVs) are small biological particles released into biofluids by every cell. Based on their size, they are classified into small EVs (<100 nm or <200 nm) and medium or large EVs (>200 nm). In recent years, EVs have garnered interest for their potential medical applications, including disease diagnosis, cell-based biotherapies, targeted drug delivery systems, and others. Currently, the long-term and short-term storage temperatures for biofluids and EVs are −80 °C and 4 °C, respectively. The storage capacity of EVs can depend on their number, size, function, temperature, duration, and freeze–thaw cycles. While these parameters are increasingly studied, the effects of preservation and storage conditions of EVs on their integrity remain to be understood. Knowledge gaps in these areas may ultimately impede the widespread applicability of EVs. Therefore, this review summarizes the current knowledge on the effect of storage conditions on EVs and their stability and critically explores prospective ways for improving long-term storage conditions to ensure EV stability.

## 1. Introduction

Extracellular vesicles (EVs) are nano-scaled particles derived from cells that aid cell-to-cell communication [1,2]. Chargaff & West (1946) alleged the presence and possible function of EVs while studying thromboplastin and platelet function [3]. The following studies also confirmed that all eukaryotes and prokaryotes could produce EVs, and it was considered that cells used these EVs as cargo transporters to transfer unwanted materials outwards [4,5,6,7,8,9,10]. However, recent research has shown that EVs are crucial for intercellular communication throughout normal and pathological development [11,12,13,14,15]; in fact, some of them are involved in cancer progression [16,17,18,19], obesity and metabolic diseases [20,21,22,23,24], inflammatory and autoimmune pathogenesis [25,26,27]. Nowadays, various studies revealed that EVs are used in several clinical applications such as disease diagnosis and development of vaccines, drug distribution, and extracellular vesicle (EV)-based therapies [17,18,19]. Electron microscopic imaging and nanoparticle tracking analysis (NTA) are the primary tools for the characterization of the morphology and size of vesicles. However, the long-term and broad-spectrum usage of EVs in clinical applications faces many obstacles. As such, preservation of their characteristics and integrity during long-term storage is critical and remains a challenging obstacle. This review summarizes the current understanding of the impact of storage conditions on EVs and their stability. It also discusses potential methods to improve long-term storage conditions to maintain EV stability.

### 1.1. Characteristics and Cargos of EV

#### 1.1.1. EV Formation and Types

Every cell secretes lipid-bound vesicles into body fluids. Based on their physical size, biogenesis, and cell surface antigens, these vesicles are classified into three major subtypes: apoptotic bodies, microvesicles (MVs), and exosomes [28]. Apoptotic bodies are EVs with a subcellular membrane mainly released by dying cells. Their diameter ranges between 50 nm and 5 µm. They may also contain intact organelles, chromatin, glycosylated proteins, proteins associated with the nucleus (histones), mitochondria (HSP60), Golgi apparatus and endoplasmic reticulum constituents (GRP78) [29,30].

**Figure 1:**
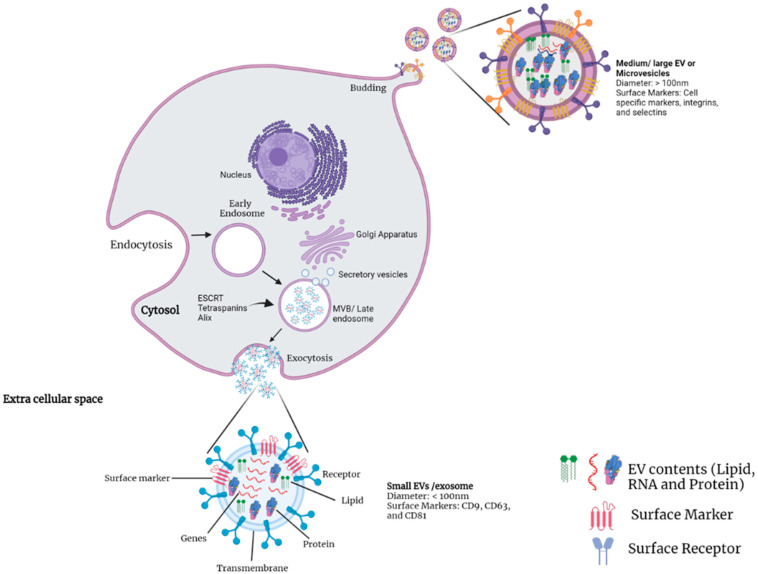
**Biogenesis of EVs and their components**. Medium EVs (mEVs) or large EVs (lEVs) are produced when the plasma membrane begins to bud. mEVs/lEVs are (>200 nm), irregular in shape, and may include cytoplasmic components. Surface markers such as integrins, CD40, selectins, and proteins from the cell are present. Small EVs (sEVs) are derived from the endosomal trafficking pathway and so have a more consistent shape and size (<200). sEVs are more readily identified than mEVs/lEVs by cell surface markers such as CD9, CD63, and CD81, and may include mitochondrial DNA, messenger RNA, and microRNA. MVB: multi-vesicular bodies; CD: cluster of differentiation. This figure was created with BioRender.com, accessed on 29 March 2022.

MVs are secreted by outward budding or pinching of the cell plasma membrane, and their diameter ranges from 100 to 1000 nm. Actin, microtubules, molecular motors (kinesins and myosins), and fusion machinery (Soluble N-ethylmaleimide-sensitive factor Attachment Protein Receptor (SNARE) and tethering factors) are believed to be needed for MVs formation. The quantity of generated MVs is dependent on the physiological conditions and microenvironment of the donor cell. Exosomes are 30–150 nm-sized single-membraned vesicles released by all cell types; they are secreted through endosomal activity and are also known as intraluminal vesicles (ILVs). Early endosomes originate from the inner budding of the cell plasma membrane, then mature into multi-vesicular bodies (MVBs). Those MVBs participate in endocytic and trafficking activities within the cell. Then, they are either destroyed in the lysosome or fused with the plasma membrane and discharged into the extracellular space [29,30,31,32,33,34,35]. The biogenesis of EVs and their components are shown in Figure 1. Due to the substantial overlap between these EV categories and the lack of consensus on specific surface markers, the use of the terminology mentioned above for EV classification is strongly discouraged. Therefore, the current guidelines were introduced by ISEV in 2018; based on the new guidelines, EVs are classified into three classes according to their physiological characteristics, i.e., (i) size—small EVs (sEVs) (<100 nm or <200 nm in diameter), medium EVs (mEVs), or large EVs (lEVs) (>200 nm in diameter)—or density—low: 1.1 to 1.2 g/mL, middle: 1.16 g/mL, or high: 1.24–1.28 g/mL—(ii) biochemical composition (using cluster of differentiation (CD)-63^+^/CD81^+^ staining, annexin V staining, etc.); (iii) cell of origin or conditions, including podocyte EVs, hypoxic EVs, large oncosomes, and apoptotic bodies [36,37,38,39]. 

#### 1.1.2. EV Membrane Components

EV membranes are composed of a lipid bilayer; some are composed of a single membrane, and the surface contains glycan and polysaccharides [40]. The presence of a bioactive cargo protecting by the lipid-bilayered membrane of EVs suggests that their molecular content could serve several therapeutic applications and their surface molecules could allow EVs to be used as biomarkers to identify different molecular subtypes [41]. However, EVs are rich in lipids, including phosphatidylcholine (PC), phosphatidylethanolamine (PE), phosphatidylserine (PS), phosphatidylinositol, phosphatidic acid, cholesterol, ceramide, sphingomyelin, glycolipids, as well as a few other lipids present in the plasma membrane [42,43]. They contain lipid-metabolizing enzymes such as phospholipases D and A2 [44]. CD-63, CD-81, major histocompatibility complex (MHC)-1, tetraspanins, growth factor receptors, integrins (ITGs), heat shock proteins, and cell adhesion molecules are membrane proteins that help to identify them [37,44,45,46]. Actin and tubulin (cytoskeleton proteins), ESCRTs (endosomal sorting complexes required for transport) protein complexes, ALIX (apoptosis-linked gene-2 interacting protein X), and tumor suppressor gene (TSG)-101 protein (inner peripheral membrane proteins) are also involved in EV structural formation [42].

In pathological conditions, affected cells release more EVs to communicate with their surroundings; these EVs are more recipient-cell-specific molecules. For example, hypoxia increases cellular communication through EV in cancer cells, mediated by hypoxia-inducible factor (HIF)-1 [47]. Cell stress stimulation and calcium ionophores may also promote vesicle release [2]. Similarly, ovarian cancer-derived EVs are uptaken by natural killer cells, whereas T cells acquire very few [48]. EVs can bind to receptor cells, or their endocytosed cargo may activate intracellular signaling cascades [49]. 

#### 1.1.3. EV Cargos

EVs carry genomic, mitochondrial deoxyribonucleic acids (DNAs), microRNA (miR), and long non-coding ribonucleic acid (lncRNA). These ribonucleic acids (RNAs) can regulate gene expression in recipient cells [50]. Then, EV-DNAs represent the whole genomic DNA of parental cells, including mutations [51]. The length of EV-RNAs is half that of cellular RNAs. MicroRNAs and transfer RNAs make up around 15% of EV-RNA extracted from serum. However, their mechanism of action in cells is unknown [52]. EVs also contain tetraspanins, cytoskeletal proteins, heat shock proteins, integrins, and other proteins. These components are mainly involved in the internalization and intracellular trafficking of EVs, followed by intracellular signaling initiation or the release of biochemical messages [53]. EV types, surface markers, and cargos are listed in Table 1.

## 2. Current Storage Conditions

EVs can be isolated from several biofluids, such as bronchoalveolar lavage fluid (BALF), seminal fluid, milk, urine, and blood. The storage of biofluids before the isolation of EVs affects the segregation, content, and function of EVs. Before isolating EVs, biofluid samples are frequently stored for short or long periods under various conditions, such as refrigeration or freezing. Isolated EVs are resuspended in phosphate-buffered saline (PBS); they are likely to be unstable at 4 °C, exhibiting a decrease in number and surface markers expression or a change in size. On the other hand, isolated EVs should be stored at −80 °C or lower. In addition, some articles recommend short-term storage ranging from a few hours to a few days at 4 °C. These storage conditions are widely used in several laboratories, most likely due to the widespread belief in EVs’ extreme stability and to reports of EV degradation following repeated freeze–thaw cycles [36,54,55,56,57,58,59]. As a result, no optimal conditions for storage have been determined, although a limited quantity of relevant data is available. These factors explain why distinct EVs would experience various modifications during storage, including physiological changes such as increased size and vesicle fusion, which result in the production of multiple vesicles in diverse shapes or a decrease in vesicle number, and functional changes such as in cargo expression. EVs may undergo various degrees of change during storage, resulting in their size, shape, function, and content loss. This review compiles data from multiple sources and outlines the impact of storage on the physiological and functional aspects of EVs.

## 3. Biofluids and Extracellular Vesicles Characterization under Different Storage Conditions

Three important factors that can frequently affect the physiological and biological characteristics of EVs are (i) temperature, (ii) pH, and (iii) preservation techniques. 

### 3.1. Impact of Temperature on Biofluids and EV during Storage

#### 3.1.1. Impact on Yield, Morphology, and Integrity

##### Storage of BALF and Their EVs

The long-term storage of EV-containing BALF can significantly disrupt the surface, morphological characteristics, and cargo proteins of BALF-EVs. It was shown that storing BALF destabilizes its surface properties, morphological characteristics, and protein content. The diameter of exosomes stored at 4 °C and −80 °C increased by 10% and 25%, respectively, compared to that of fresh exosomes. The proteomic content of the EVs was also lost due to storage at 4 °C and −80 °C [55]. 

##### Storage of Urine, Sperm, and Their EVs

Zhou et al. (2006) demonstrated that storing urine with protease inhibitors inhibited exosome-associated protein degradation (sodium–hydrogen exchanger 3 (NHE3), TSG101, ALIX, and aquaporin 2 (AQP2)). Urine samples were stored at −80 °C for seven months, and the expression of exosome-associated NHE3, TSG101, ALIX, and AQP2 proteins was better preserved than in samples stored at −20 °C [60]. Human urine was collected and stored at 4 °C and −80 °C for 24 h. Then, the expression of the TSG101, AQP2, angiotensin-converting enzyme, and podocalyxin-like (PODXL) proteins was analyzed and compared to that in fresh urine; the expression of the above markers was stable after storage [61]. Though EVs present in fresh urine samples may decrease their size within 2 h of collection, the optimal storage of urine samples containing EV can be achieved by storing them at −80 °C in a solution of 0.5 mM phenylmethylsulphonyl fluoride (PMSF) and 20 mM leupeptin (final concentration 1:10) as a protease inhibitor for a week [56]. In contrast, long-term freezing does not affect the protein and mRNA levels of CD63 and CD9. Briefly, freezing sperm for two years at −80 °C had no discernible effect on the biological activity of EVs, regardless of their size, structure, or concentration [57,58]. In another study, the collected cell-free urine was stored in freezing conditions for one year; then, the EVs were collected and compared with EVs collected from fresh urine. The concentration of EVs isolated from the fresh urine sample was 10^9^–10^10^/mL; this concentration decreased 2-fold after a single freeze–thaw cycle. The diameter of EVs increased by 17% after storage. However, there were no morphological changes observed after storage [59]. Overall, these studies recommend that storage at −80 °C with protease inhibitors is suitable to preserve EV-containing urine samples for long periods. 

##### Storage of Milk and Their EVs

Munagala et al., 2016, discovered that EV-containing bovine milk stored at −80 °C for several months without coagulation retained a high degree of activity [62]. Another study reported that EV-containing bovine milk was stored at −80 °C for 28 days without affecting the physiological characteristics of the EVs [63]. Unrefined human breast milk stored at 4 and −80 °C showed cell death and storage stress, which can affect the EV population and a minor effect on the expression of the CD-63 and CD-9 EV markers [64]. EV-containing bovine and human milk can be stored at −80 °C for 28 days with minimal physiological characteristic loss.

##### Storage of Blood, Plasma, Serum, and Their EVs

Blood, serum, plasma, and platelets are vital fluids used for EV isolation. In contrast to other biofluids, long-term storage and freeze–thaw cycles of serum do not affect EV yield. For example, human serum EVs remained stable under various conditions, including storage at room temperature (RT) for 24 h, at 4 °C for seven days, at −80 °C for one year, and under certain repeated freeze–thaw cycles [65,66]. However, fresh plasma yielded more and purer and EVs than stored plasma [65].

The short-term storage of human blood at RT or 4 °C resulted in an increase in the expression of the surface markers CD9, CD63, and CD81 compared to long-term freezing storage [67]. Human plasma was collected from healthy people and stored at 4 °C for 7 h and at −80 °C for 7 and 28 days. Storage at 4 °C did not affect the number of endothelial microparticles present. However, storage at −80 °C enhanced the expression of CD31+, CD42b−, and CD62E+ and decreased the expression of CD144+ [68]. Another study reported that EV concentration decreased significantly in plasma stored at −80 °C for 10–12 days compared to freshly isolated plasma samples. However, no significant difference was observed in the average size of EVs between the samples [69]. Platelets were frozen and cryopreserved for 24 h at −80 °C in 5–6% dimethyl sulfoxide (DMSO). More microvesicles were released, which a significant increase over the 24 h short-term storage period, than from fresh platelets [70]. Storage of red blood cell at 4 °C for 50 days increased the particle counts by 20-folds and increased protein expression [71]. However, storage at −80 °C for 6 months of plasma before EV isolation and of isolated EVs was associated with a decrease in particle concentration and an increase in protein content and particle size. The freeze–thaw process also lowered the yield, increased particle size, and triggered membrane breakdown and re-micellization [72]. Meanwhile, Jin et al., 2016 reported that serum-derived exosomes stored at RT showed significant changes in CD63, TSG101, and DNA concentrations after 24 h. In addition, the increased stability of isolated exosomes stored at 4 °C for seven days, without effects on CD63, TSG101, or nucleic acid concentrations, was observed [65]. Based on these findings, EVs extracted from blood, and their derivatives, can be stored at 4 °C for two weeks or frozen for two years.

Sokolova et al. (2011) stated that the maintenance of EVs’ integrity and size highly depend on the storage conditions [73]. The storage of EVs at 4 °C for 24 h decreased their population, while the physical and functional characteristics of EVs were preserved for 28 days at −80 °C [74]. A single freeze–thaw cycle and storage in frozen conditions such as at −20, −80, and −196 °C did affect the concentration, diameter, and the expression of the surface markers CD235a+ and CD61+ of human plasma-derived EVs for up to one year. At the same time, lactadherin+ expression in EVs increased sevenfold compared to that in fresh plasma samples [59]. Deville et al. (2021) revealed no differences in number between freshly isolated EVs and EVs stored at 4 or −80 °C for up to one month [75]. These studies showed that the particle concentration and size distribution and the concentrations of erythrocyte and platelet EVs in plasma were relatively stable following a single freeze–thaw cycle and storage for up to one year. However, the average size of the exosomes increased after four days of storage at 4 °C. Freeze–thaw cycles significantly decreased exosome concentration, quality, and protein level [55]. 

##### Storage of Saliva

Yuana et al. (2015) collected saliva from healthy humans and stored it in frozen conditions at −20, −80, and −196 °C for one year. Then, EVs were isolated. Compared to EVs isolated from fresh samples, the diameter of these EVs increased by 17%, and their concentration decreased 3-fold after a single freeze–thaw cycle and storage. Nevertheless, there no morphological changes were observed [59]. Human saliva was stored at 4 °C for 7 days; after that, the total protein, dipeptidyl peptidase IV activity, morphology, and expression of exosomes’ surface markers (CD9, ALIX, and TSG101) were stable, but minor evidence of degradation of certain proteins was also found after storage [76].

##### Storage of EVs from Cell Culture Media

Lee et al. (2016) demonstrated that exosomes stored for 10 days at RT had reduced expression of the exosomal marker CD-63 but not of CD-9, RNA, and proteins, as well as no reduction in the population, compared to exosomes preserved at 4 °C and −70 °C and fresh exosomes. Exosome uptake efficiency and biodistribution were significantly decreased after storage at 4 °C and −20 °C [77]. However, exosomes stored at −80 °C for 14 days maintained their biodistribution expression [78]. Park et al. (2018) explained that the size, number of EVs, surface protein expression (CD-63 and -81), and functional stability remained constant at −70 °C after 25 days [79]. Nevertheless, Richter et al. (2019) reported that EV storage at −80 °C for 14 days altered the morphology and particle size and increased particle aggregation [80]. Cheng et al. (2019) observed no changes in the concentration of exosomes and exosomal markers (ALIX, TSG101, and heat shock protein (HSP)-70) expression after 24 h of storage at 4 °C [81]. These studies proved that −80 °C is the optimal temperature for long-term EV storage (up to 28 days). Long-term or short-term storage under frozen conditions or refrigeration of biofluids or EVs slightly affects EVs’ morphological characteristics and surface marker expression.

#### 3.1.2. Impact on Functional Activities and Cargos Expression

EVs storage at −80 °C for 14 days decreased the glucuronidase activity [80]. Human blood serum was incubated at RT for up to 24 h before isolating EVs. Then, exosomal miRNA expression was compared to that of freshly isolated EVs. It was found that miR-21 and miR-142-3p expression was reduced compared to freshly prepared EVs [82]. At the same time, another study reported that after storage of human blood serum at 4 and −70 °C for long periods, the expression of miRNAs such as miR-21, miR-200b, and miR-205 in isolated exosomes was stable for up to 96 h at 4 °C and 28 days at −70 °C [83]. Another study confirmed that the Ct values of exosomal lethal (let)-7a and miR-142-3p were stable after storing human blood plasma at 25 °C for 48 h [84]. In addition, Ge et al. (2014) isolated exosomes from plasma and kept them in the freezer for two years, then showing stable exosomal miRNA, with no detectable changes. Nonetheless, two weeks of storage at 4 °C may decrease exosomal miRNA levels [85]. In contrast, Baddela et al. (2016) stored buffalo milk at 4 °C for 24 h before exosome isolation. The expression of exosomal miR-21 was two-fold lower than that in fresh milk exosomes [86]. 

Madison et al. (2015) demonstrated that EVs isolated from human seminal fluid play an essential role in human immunodeficiency virus (HIV)-1 transformation. However, 30 years of prolonged freezing of seminal fluid at −80 °C before isolation of EVs decreased the capability of EVs to inhibit HIV-1 infection in cells via a decline of acetylcholine-esterase (AChE) activity. However, the small sample size of this study limits the interpretation of the observed results and the conclusion of this study. A larger sample size is necessary to overcome these constraints and make conclusions about the exosome phenotypes and their relationship to AChE, CD63, CD9, and HIV-1 [57]. This study proved that the long-term storage of EV-containing seminal fluid at −80 °C is possible, with minimal activity loss. It was shown that long-term storage, from 28 days to 12 years, at RT or −80 °C and two freeze–thaw cycles did not significantly degrade EV-associated RNAs [83,85,87]. At the same time, another study found that annexinV^+^ EV expression was stable in plasma samples following a single freeze–thaw cycle, but it was decreased in samples stored at −80 °C. After seven years of −80 °C storage, protein and nucleic acid aggregation occurred [88,89]. 

### 3.2. Impact of pH

Some studies also confirmed that pH could affect EVs’ characteristics and preservation. Nakase et al. (2021) demonstrated that a low pH (pH 5) in cell culture affected EV production by increasing their protein content and zeta potential. Interestingly, a low pH also increased EVs uptake into recipient cells [90]. Storing exosomes in acidic (pH 4) or alkaline (pH10) conditions increased the aggregation of exosomes as well as exosome uptake by cells when compared to storage at pH 7 [81]. Zhao et al. (2017) reported that incubating exosome-associated proteins for 30 min at RT at an acidic pH < 7 inhibited their degradation and increased the yield of exosomes in a conditioned medium or urine. Another study investigated the impact of acidic conditions on EV functions and discovered that massive exosome-associated doxorubicin was released more rapidly at pH 5 than at pH 7.4 [91]. Macrophage-derived EVs are degraded by 90% in an acidic environment [92]. Overall, acidic conditions were found to increase exosome release and uptake.

### 3.3. Impact of Preservation Techniques

The major preservation techniques include cryopreservation, freeze–drying, and spray–drying and are involved in EVs storage [93]. 

Cryopreservation is based on the use of low temperatures to maintain EVs function. Different cryoprotectants are used to protect EV effectiveness at low temperature. Two types of cryoprotectants are available, i.e., penetrating cryoprotectants and non-penetrating cryoprotectants. Penetrating cryoprotectants (e.g., glycerol, DMSO, and ethylene glycol) can enter the cell and preserve it during the freezing process [94]. Tegegn et al., 2016, demonstrated that platelet samples stored in frozen conditions using 6% DMSO as a cryoprotectant produced more EVs and retained their procoagulant activity when compared to platelets stored at RT [95]. Besides being added after EVs isolation, 10% DMSO can be added to cryopreserved samples before EVs isolation to counteract the decline of EV RNA [96]. Non-penetrating cryoprotectants (e.g., sucrose, mannose, and trehalose) can form hydrogen bonds with water, reducing the damage to EVs [94]. According to Gelibter et al., 2022, after six months of storage at −80 °C, there was no significant reduction in EV concentrations when EVs were stored with or without various cryoprotectants such as trehalose 25 mM, DMSO 6 and 10%, glycerol 30%, protease inhibitors, and sodium azide at 4 °C or after lyophilized with trehalose [72]. However, different cell-derived EVs showed other features after storage. 

Freeze–drying is a two-step method that includes sublimation and desorption. Freeze–drying is an emerging technique for preserving EVs, and 4 °C is the optimal storage temperature for freeze–dried EVs [97,98]. When EVs produced from cerebrospinal fluid (CSF) were lyophilized and held at RT for seven days, EV number decreased by 37–43%. In addition, this reduction was related to a decrease in the abundance of representative miRNAs. By contrast, the number and shape of EVs remained virtually constant in these conditions. In this context, total RNA and representative miRNA levels were stable for up to seven days. A single cycle of freezing and thawing had no discernible influence on the number of EVs, their shape, RNA content, or miRNA levels. However, after two freezing and thawing cycles, these characteristics gradually declined [99]. 

EVs were isolated from mesenchymal stem cells (MSC), HUVEC, and A549 cells. Collected EVs were stored at −80 °C, 4 °C, RT, or lyophilized for 2 and 14 days. After two days of storage, lyophilization did not affect the size of EVs. However, the concentration of A549 EVs decreased, while the concentration of MSC EVs remained constant. Storage for 14 days did not change MSC EV yield, but the particle size non-significantly increased after lyophilization. When HUVEC EVs were lyophilized, the particle number non-significantly decreased after 14 days of storage, while the size of the particles was not affected. The size of A549 EVs did not change significantly during lyophilization. However, the particles’ concentration decreased [100]. He-La cells EVs were lyophilized, and the particle size was not altered, as it remained in the range from 76.7 ± 22.5 to 85.9 ± 38.7. The zeta potential was almost the same, −10 mV, before and after lyophilization. It affected the cytosolic release efficacy of the EV content after cellular uptake. Therefore, lyophilization did not affect the structure and characteristics of EVs. However, it affected their function [101]. Richter et al. (2019) demonstrated that after storage of HUVEC-derived EVs at 4 °C, −80 °C and lyophilization with 4% trehalose for seven days, the mean size of the particles increased slightly, while glucuronidase activity and the percentage of particle recovery decreased. Compared to storage at 4 °C and −80 °C, freeze–drying with trehalose resulted in the least amount of loss of HUVEC-EV characteristics [80]. EVs from human adipose-derived stem cells were lyophilized with trehalose or trehalose/polyvinylpyrrolidone 40 (PVP40) as lyoprotectants. After 24 h, there was no significant alteration in particle number, size, and function [102]. Generally, freeze–drying is a cost-effective method for storing EVs at RT, not or slightly affecting their morphology or cargos. It could also be used to increase the lifespan of EVs. 

Spray–drying is a single-step method that is easier than freeze–drying; it can be used with various agents and allows adjusting the size of the products. The EV solution is first atomized, and the heated gas powders these droplets; these are fully automated processes. The rate of EV solution feed, atomization pressure, and outlet temperature affect the EVs and their cargo stability. Additionally, residual moisture may increase the chemical instability by lowering the glass transition temperature of the solid particle state. Additional research is required to apply this technique to the production of EV-based therapeutics [103]. Table 2 and Table 3 summarize the storage conditions and their impact on EV-containing biofluids and isolated EVs.

**Figure 2:**
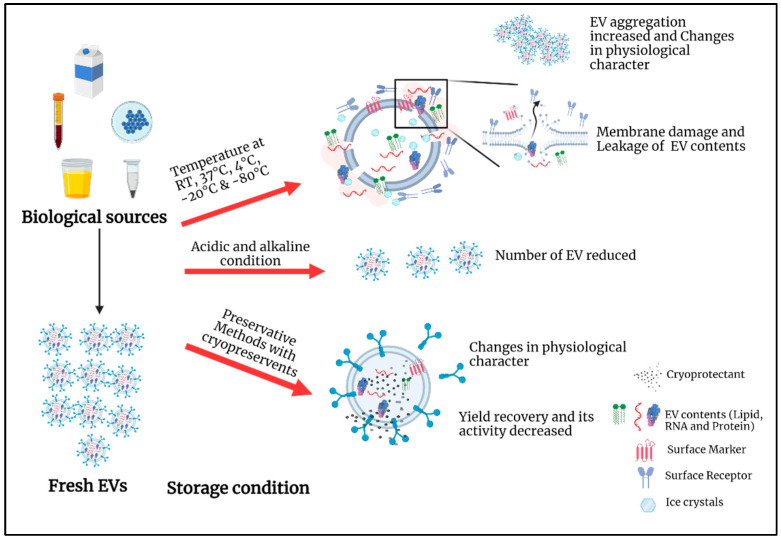
**Impact of storage conditions on EVs**. Various EV storage conditions and their potential effects on EV integrity. Biological sources or freshly isolated EVs are suspended in PBS with or without cryopreservants. Then, they are stored in specific conditions. The prolonged storage at low temperature induces mechanical damage, resulting in a loss in membrane integrity, leakage of EV cargo (RNA, protein, and lipids), and detachment of EV surface molecules (receptors and markers) due to the formation of tiny ice crystals within as well as around the EVs; Acidic or alkaline conditions reduce the number of EVs. Preservation methods also change the physiological properties of EVs and facilitate their product recovery. This figure was created with BioRender.com.

Some researchers warned that isolated EVs could be adsorb onto tube walls, which would decrease EV concentration. Using Eppendorf Protein LoBind tubes or adding bovine serum albumin or Tween-20 to block the wall can decrease the loss [104,105]. Hermida-Nogueira et al. (2020) found that seven days following a pathogen reduction technology (PRT) + Mirasol^®^ (vitamin B2 with ultraviolet B light), 151 proteins, including EV markers and regulatory proteins released by platelets, were upregulated compared to their levels after two days of storage [106]. Sources of EVs, storage conditions, and their impact are presented in Figure 2.

## 4. Prospects

EV productivity and storage condition improvement are essential to promote basic research and therapeutic applications. Consequently, determining optimal EV storage conditions is necessary for storing, transporting, and maintaining EV quality, preserving EV functions, and enhancing therapeutic outcomes. Currently, EVs can be stored at 4 °C without freeze–thaw cycles for short times (a day or a few weeks), and a temperature of −80 °C is recommended with a few freeze–thaw cycles for long-term (months or years) storage [36,38,54]. During frozen storage, high concentrations of EVs can promote vesicle interactions and promote damage. Sometimes, differential centrifugation also causes particle aggregation. Exploring the optimal storage conditions of EVs from a temperature perspective is not sufficient because ice crystal formation is also related to the cooling rate. In addition, Freeze–thaw cycles may also damage EVs, which are fragile when suspended in phosphate-buffered saline. However, prolonged storage at freezing temperatures and repeated freeze–thaw processes may affect the EVs’ characteristics. Detailed research is required; adding some exogenous compounds can potentially overcome this difficulty and protect EVs’ characteristics. These substances are referred to as “cryo-preservative agents (CPA).” Additionally, CPA aid in achieving optimal EV dehydration by increasing viscosity, regulating ice nucleation kinetics, and promoting extracellular ice growth during temperature reduction. 

A nonreducing natural disaccharide has been approved by the FDA for use as a CPA to store a variety of proteins and cellular products [107]. Moreover, trehalose is less water-soluble than sucrose (except at temperatures above 80 °C), has high water retention capacity, and its anhydrous forms rapidly reclaim moisture to form the dihydrate [108,109,110]. When stored at RT, trehalose inhibits exosome aggregation and exhibits the same pharmacokinetic profiles in mice [98]. In another study, mannitol was shown to protect exosomes during lyophilization and storage at −20 °C [111]. Freeze–drying is a cost-effective method for storing EVs at RT, with no effects on their morphology or materials. Carried out with a CPA concentration of less than 0.1 M and a cooling rate of around 1 °C/min. Apart from CPAs, additional cryopreservation methods are vitrification or slow cooling cryopreservation. On the other hand, introducing programmed cooling into EV cryopreservation may be an excellent strategy to improve the integrity and functions of EVs [42,103,112,113]. 

## 5. Conclusions

According to current evidence, one of the most common short-term storage temperatures for EVs is 4 °C, Also, −80 °C is the best for long term storage of EV containing biofluids and isolated EVs. However, these storage conditions have a significant impact on the integrity and functions of EVs; they are also expensive and cause difficulties in EV transport. Further investigations are required to optimize the current storage conditions and develop advanced techniques for preserving the quality, integrity, and functions of EVs.

## Figures and Tables

**Table 1:** EV types, surface markers, and cargos.

Features	Apoptotic Bodies	MVs	Exosomes
Shape	Heterogeneous	Heterogeneous	Spherical
Size (nm)	50–5000	100–1000	30–150
Formation mechanism	Nuclear chromatin condensation, followed by membrane blebbing	Plasma membrane direct outward budding and fission	Endosomal network fusion with the plasma membrane
Release or response	Apoptosis	Cell injury, proinflammatory stimulants, hypoxia, oxidative stress or shear stress	Cellular stress or activation signals
Surface markers	Apoptotic cell markers	Selectins, integrin, CD40, CD31+, CD235a+, CD42b−, CD45, CD61+, CD62E+, and CD144+,	Tetraspanins (CD9, CD63 CD81 and CD82)
Cargos and other markers	Intact chromatin, glycosylated proteins, Caspase 3, histones, HSP60, and GRP78	Cytoskeletal proteins, heat shock proteins, integrins, and proteins containing post-translational modifications, such as glycosylation and phosphorylation	ALIX, TSG-101, PODXL, HSP70, and HSP90β

**Table 2:** Summary of the impact of storage conditions on EV-containing biofluids.

Source	Type of EVs	Storage Temperature/pH/Cryopreserves	Duration	Freeze-Thaw Cycles	Physical Changes	Functional Changes	References
**BALF**	EV	−80 °C	4 days	-	Disruption in the surface and morphological characteristics and ↓ total protein content	-	[55]
**Serum**	MP	−80 °C	1 week and 1 year	1	Microparticle counts are stable	-	[66]
	EV	RT	24 h	-	-	↓ miR-21 and miR-142-3p	[82]
	EV	4 and −70 °C	96 h and 28 days	-	-	miR-21, miR-200b, and miR-205 expression was stable	[83]
**Blood**	exosomes	RT, 4 °C, −20 °C, −80 °C and −160 °C	Days and Months	-	-	The stable expression in signals under storage at RT and 4 °C for long-term storage. ↓ signal intensities for long-term storage	[67]
**Plasma**	Microparticles	4 °C and −80 °C	7 h, 7 and 28 days	-	↑ expression of CD31+, CD42b- and CD62E+ ↓ expression of CD144+	-	[68]
	EV	−80 °C	10–12 days	-	↓ EV concentration No changes in EV size	-	[69]
	EV	−80 °C	6 months	-	↓ Particle concentration ↑ Total protein content and particle size	-	[72]
	EV	−80 °C	12 months	1	-	↓ level of AnnV^+^ before thaw; and ↑ level of AnnV^+^ after a single freeze-thaw cycle	[88]
	Exosomes	RT	0–48 h	-	-	Ct value of exosomal let-7a and miR-142-3p were stable	[84]
		−80 °C	7 years	-	-	↑ Amount of total protein and protein/nucleic acid aggregation	[89]
**Platelets**	MV	−80 °C	24 h	-	↑ MV secretion	-	[70]
	EV	pathogen reduction technology (PRT) treatment with Mirasol® (vitamin B2plus UVB light)	2 and 7 days	-		↑151 proteins, including EV markers	[103]
	EVs	Frozen with 6% DMSO		-	↑ EV production	Procoagulant activity was stable	[95]
**RBC**	EV	4 °C	50 days	-	↑ 20-folds Particle counts	-	[71]
**Milk**	EV	4 °C and −80 °C	2–8 weeks	-	-	No changes in CD63 and CD9 expression	[64]
	Exosomes	−80 °C	4 week	-	↑ contamination by stress-induced exosomes	-	[63]
			6 month	-			[62]
	EV	4 °C	24 h	-	-	↓ 2-fold-miR-21 expression	[86]
**Urine**	Exosomes	−20 °C & −80 °C with PI	1 week	-	↓ EV associated protein expression	-	[60]
		4 °C and −80 °C	24 h	-	Stable expression of TSG101, AQP2, angiotensin-converting enzyme, and PODXL	-	[61]
		RT, 4 °C and −80 °C	2 h–7 days	-	↓ EV yield	-	[56]
**Semen**	Exosomes	−80 °C	2 and 30 years	-	Size, structure, or concentration are stable	↓ Amount of protein, AChE, and anti-HIV activities on long-term freezing. But total RNA level is stable	[57,58]
**Saliva**	EV	−80 °C	1 year	1	↓ 2-fold EV concentration, ↑ 17% in size, Morphological characteristics are stable.	-	[59]
	Exosomes	4 °C	7 days	-	No changes in total protein, dipeptidyl peptidase IV activity, morphology, and surface markers (CD9, ALIX, and TSG101)	Degradation in some functional proteins	[76]
**A431 cells (Culture media)**	EVs	pH 5, 6 and 7 (cell culture condition)	24 h		pH 5 cell culture condition increases its protein content and zeta potential.	↑ EV uptake into recipient cells	[90]
**HEK293T cells** **(Culture media)**	EV	RT, 4 °C, −20 °C and −80 °C	10 days	-	↓ CD63 expression under storage at RT and 4 °C. More stable in protein and RNA expression under storage at frozen condition	Exosome uptake efficiency and biodistribution were significantly decreased when stored at 4 °C and −20 °C	[77]
	Exosomes	60 °C, 37 °C, 4 °C, −20 °C, and −80 °C at pH 4, 7, or 10	1 day	2	No changes occur in ALIX, HSP70, and TSG101 at 4 °C. ↓ Exosome numbers in pH 4 and 10.	↑ Cellular uptake of exosomes at pH4 and 10. ↓ Cellular uptake under stored at 4 °C	[81]
**HUVEC** **(Culture media)**	EV	37.4, −20, and −70 °C	25 days	-	↓ particle number and ↑ size on 37.4, and −20 °C	↓ CD-63 and -81 expression under storage at 37 °C. ↓ Functional stability on 37 and −20 °C	[79]
**THP-1** **(Culture media)**	EV	4 °C and −80 °C	1 week, 2 weeks, or 1 month	-	Stable EV concentration on all temperature	-	[75]
**b. End.3 cells** **(Culture media)**	Exosomes	4 °C, −20 °C, and −80 °C	0–28 days	1–5	↓ particle number and ↑ size under all storage conditions. ↓ Number of exosomes for all freezing conditions	↓ Amount of protein, RNA, and uptake efficiency at 4 °C.	[78]
**CSF**	EVs	Lyophilized and held at RT	7 days	2	↓37–43% in EV number. The shape of the EV is not stable	↓ miRNAs abundance	[99]

↑—Increased; ↓—Decreased; A431 cells—Cellosaurus cell line; AchE—acetylcholine-esterase; ALIX—Apoptosis-linked gene 2–interacting protein X; AQP— Aquaporin 2; BALF—Bronchoalveolar lavage fluid; b. End.3—Brain endothelial-3 cells; CD—Cluster differentiation; CSF—cerebrospinal fluid; DNA—Deoxyribonucleic acid; EV—Extracellular vesicle; HEK293T—Human embryonic kidney 293 cells; HIV—Human immunodeficiency virus; HUVEC—Human umbilical vein endothelial cells; MP—Microparticle; miR—MicroRNA; MV— Microvesicle; PODXL—Podocalyxin-like protein; PI—Protease Inhibitor; RT—Room temperature; PRT—pathogen reduction technology; RBC—Red blood cells; RNA—Ribonucleic Acid; THP-1—human leukemia monocytic cell line; TSG—Tumor susceptibility gene and UVB—Ultraviolet B.

**Table 3:** Summary of the impact of storage conditions on isolated EVs.

Source	Type of EVs	Storage Temperature/pH/Cryopreserves	Duration	Freeze-Thaw Cycles	Physical Changes	Functional Changes	References
**BALF**	Exosomes	4 °C, and −80 °C	4 days	-	↑ Size of exosome	↓ protein concentration	[55]
**Plasma**	EV	4 °C, −20 °C & −80 °C	2 weeks–2 years	-	-	↓ RNA or protein expression, storage at 4 °C for 2 weeks. No changes in RNA or protein expression storage at −80 °C	[85]
**Serum**	EV	RT and 4 °C	6 h–1 week	1, 3, and 5	-	No changes occur in CD63, TSG101, expression, and DNA concentration at RT storage for 24 h; and 4 °C for 1 week. ↓ DNA concentration due to freeze-thaw cycles but no changes in CD63 and TSG101 expression	[65]
**He-La cells** **(Culture media)**	EV	Lyophilization	48 h	-	Particle size and zeta potential stable	-	[101]
**MSC (Culture media)**	EV	−80 °C, 4 °C, RT, or lyophilized	2–14 days	-	↑ Particle size by −80 °C, 4 °C, RT ↑ Particle size non-significantly by lyophilization	-	[100]
**A549 cells** **(Culture media)**	EV	−80 °C, 4 °C, RT, or lyophilized	2–14 days	-	↓ Particle concentration and Particle size stable after lyophilization	-	
**HUVEC** **(Culture media)**	EV	−80 °C, 4 °C, RT, or lyophilized	2–14 days	-	↓ Particle concentration non-significantly after lyophilization	-	
**HUVEC** **(Culture media)**	EV	4 °C, −80 °C and lyophilized with 4% trehalose	14 days	-	↑ size and % of particle recovery under all storage	↓ Glucuronidase activity	[80]
**Human Adipose-Derived Stem Cells (Culture media)**	EV	lyophilized with trehalose or trehalose/PVP40	24 h	-	Particle number and size are stable	-	[102]
**Plasma**	EV	−80 °C	6 months	2	↓ Particle concentration ↑ Total protein content and particle size	-	[73]
**Plasma**	EV	−80 °C with trehalose 25 mM, DMSO 6 and 10%, glycerol 30%, PI and sodium azide at 4 °C or lyophilization with trehalose	6 months	-	stable EV concentrations	-	[72]
**Neutrophilic granulocytes**	EV	4 °C	24 h	-	No changes in physiological characteristics	No changes in functional characteristics	[74]
**Conditioned media/Urine**	Exosomes	pH < 7 at RT	30 min	-	↑Yield and ↓ Degradation	↑ Exosome-associated doxorubicin at pH 5	[91]

↑—Increased; ↓—Decreased; ALIX—Apoptosis-linked gene 2–interacting protein X; b.End.3 cells—Brain endothelial-3 cells; BALF—Bronchoalveolar lavage fluid; CD—Cluster differentiation; EV—Extracellular vesicle; HEK 293 cells—Human embryonic kidney 293 cells; HSP—Heat shock protein; HUVEC—Human umbilical vein endothelial cells; MSC—Mesenchymal stem cells; MV—Microvesicle; PI—Protease Inhibitor; RT—Room temperature; RNA—Ribonucleic Acid; and TSG—Tumor susceptibility gene.

## Data Availability

Not applicable.

## References

[1] Tkach M., Théry C. (2016). Communication by Extracellular Vesicles: Where We Are and Where We Need to Go. Cell.

[2] El Andaloussi S., Mäger I., Breakefield X.O., Wood M.J.A. (2013). Extracellular Vesicles: Biology and Emerging Therapeutic Opportunities. Nat. Rev. Drug Discov..

[3] Chargaff E., West R. (1946). The biological significance of the thromboplastic protein of blood. J. Biol. Chem..

[4] Bazzan E., Tinè M., Casara A., Biondini D., Semenzato U., Cocconcelli E., Balestro E., Damin M., Radu C.M., Turato G. (2021). Critical Review of the Evolution of Extracellular Vesicles’ Knowledge: From 1946 to Today. Int. J. Mol. Sci..

[5] Trams E.G., Lauter C.J., Salem N., Heine U. (1981). Exfoliation of membrane ecto-enzymes in the form of micro-vesicles. Biochim. Biophys. Acta.

[6] Mishra N.C., Tatum E.L. (1973). Non-Mendelian Inheritance of DNA-Induced Inositol Independence in *Neurospora*. Proc. Natl. Acad. Sci. USA.

[7] Fox A.S., Yoon S.B., Gelbart W.M. (1971). DNA-Induced Transformation in *Drosophila:* Genetic Analysis of Transformed Stocks. Proc. Natl. Acad. Sci. USA.

[8] Fox A.S., Yoon S.B. (1970). DNA-Induced Transformation in *Drosophila:* Locus-Specificity and the Establishment of Transformed Stocks. Proc. Natl. Acad. Sci. USA.

[9] Aaronson S., Behrens U., Orner R., Haines T.H. (1971). Ultrastructure of intracellular and extracellular vesicles, membranes, and myelin figures produced by *Ochromonas danica*. J. Ultrastruct. Res..

[10] Wolf P. (1967). The Nature and Significance of Platelet Products in Human Plasma. Br. J. Haematol..

[11] Quynh N.T., Nhan L.T.T., Phuong L.L., Thao B.P., Linh N.T.T., Tho L.T., Thai T.H. (2020). Mitochondrial A10398G Alteration in Plasma Exosome of Non-small Cell Lung Cancer Patients. VNU J. Sci. Med. Pharm. Sci..

[12] Zomer A., Maynard C., Verweij F.J., Kamermans A., Schäfer R., Beerling E., Schiffelers R.M., de Wit E., Berenguer J., Ellenbroek S.I.J. (2015). In Vivo Imaging Reveals Extracellular Vesicle-Mediated Phenocopying of Metastatic Behavior. Cell.

[13] Bronisz A., Wang Y., Nowicki M.O., Peruzzi P., Ansari K.I., Ogawa D., Balaj L., De Rienzo G., Mineo M., Nakano I. (2014). Extracellular Vesicles Modulate the Glioblastoma Microenvironment via a Tumor Suppression Signaling Network Directed by miR-1. Cancer Res..

[14] Languino L.R., Singh A., Prisco M., Inman G.J., Luginbuhl A., Curry J.M., South A.P. (2016). Exosome-mediated transfer from the tumor microenvironment increases TGFβ signaling in squamous cell carcinoma. Am. J. Transl. Res..

[15] Boelens M.C., Wu T.J., Nabet B.Y., Xu B., Qiu Y., Yoon T., Azzam D.J., Victor C.T.-S., Wiemann B.Z., Ishwaran H. (2014). Exosome Transfer from Stromal to Breast Cancer Cells Regulates Therapy Resistance Pathways. Cell.

[16] He E.A.C., Li L., Wang L., Meng W., Hao Y., Zhu G. (2021). Exosome-mediated cellular crosstalk within the tumor microenvironment upon irradiation. Cancer Biol. Med..

[17] Zheng P., Chen L., Yuan X., Luo Q., Liu Y., Xie G., Ma Y., Shen L. (2017). Exosomal transfer of tumor-associated macrophage-derived miR-21 confers cisplatin resistance in gastric cancer cells. J. Exp. Clin. Cancer Res..

[18] Su T., Zhang P., Zhao F., Zhang S. (2021). Exosomal MicroRNAs Mediating Crosstalk Between Cancer Cells With Cancer-Associated Fibroblasts and Tumor-Associated Macrophages in the Tumor Microenvironment. Front. Oncol..

[19] Chen Q., Li Y., Gao W., Chen L., Xu W., Zhu X. (2021). Exosome-Mediated Crosstalk Between Tumor and Tumor-Associated Macrophages. Front. Mol. Biosci..

[20] Li C.-J., Fang Q.-H., Liu M.-L., Lin J.-N. (2020). Current understanding of the role of Adipose-derived Extracellular Vesicles in Metabolic Homeostasis and Diseases: Communication from the distance between cells/tissues. Theranostics.

[21] Akbar N., Azzimato V., Choudhury R.P., Aouadi M. (2019). Extracellular vesicles in metabolic disease. Diabetologia.

[22] Jayabalan N., Nair S., Nuzhat Z., Rice G.E., Zuniga F.A., Sobrevia L., Leiva A., Sanhueza C., Gutiérrez J.A., Lappas M. (2017). Cross Talk between Adipose Tissue and Placenta in Obese and Gestational Diabetes Mellitus Pregnancies via Exosomes. Front. Endocrinol..

[23] Ashrafian F., Shahriary A., Behrouzi A., Moradi H.R., Raftar S.K.A., Lari A., Hadifar S., Yaghoubfar R., Badi S.A., Khatami S. (2019). Akkermansia muciniphila-Derived Extracellular Vesicles as a Mucosal Delivery Vector for Amelioration of Obesity in Mice. Front. Microbiol..

[24] Pardo F., Villalobos-Labra R., Sobrevia B., Toledo F., Sobrevia L. (2018). Extracellular vesicles in obesity and diabetes mellitus. Mol. Asp. Med..

[25] De Toro J., Herschlik L., Waldner C., Mongini C. (2015). Emerging roles of exosomes in normal and pathological conditions: New insights for diagnosis and therapeutic applications. Front. Immunol..

[26] Buzás E.I., György B., Nagy G., Falus A., Gay S. (2014). Emerging role of extracellular vesicles in inflammatory diseases. Nat. Rev. Rheumatol..

[27] Zhang B., Zhao M., Lu Q. (2021). Extracellular Vesicles in Rheumatoid Arthritis and Systemic Lupus Erythematosus: Functions and Applications. Front. Immunol..

[28] Nielsen T.B., Nielsen M., Handberg A. (2014). Third International Meeting of ISEV 2014: Rotterdam, The Netherlands, April 30^th^–May 3^rd^, 2014. J. Extracell. Vesicles.

[29] Doyle L., Wang M. (2019). Overview of Extracellular Vesicles, Their Origin, Composition, Purpose, and Methods for Exosome Isolation and Analysis. Cells.

[30] Borges F.T., Reis L.A., Schor N. (2013). Extracellular Vesicles: Structure, Function, and Potential Clinical Uses in Renal Diseases. Braz. J. Med. Biol. Res..

[31] Chivero E.T., Dagur R.S., Peeples E.S., Sil S., Liao K., Ma R., Chen L., Gurumurthy C.B., Buch S., Hu G. (2021). Biogenesis, physiological functions and potential applications of extracellular vesicles in substance use disorders. Cell. Mol. Life Sci..

[32] Latifkar A., Hur Y.H., Sanchez J.C., Cerione R.A., Antonyak M.A. (2019). New insights into extracellular vesicle biogenesis and function. J. Cell Sci..

[33] Juan T., Fürthauer M. (2018). Biogenesis and function of ESCRT-dependent extracellular vesicles. Semin. Cell Dev. Biol..

[34] Bebelman M.P., Smit M.J., Pegtel D.M., Baglio S.R. (2018). Biogenesis and function of extracellular vesicles in cancer. Pharmacol. Ther..

[35] Latifkar A., Cerione R.A., Antonyak M.A. (2018). Probing the mechanisms of extracellular vesicle biogenesis and function in cancer. Biochem. Soc. Trans..

[36] Théry C., Witwer K.W., Aikawa E., Alcaraz M.J., Anderson J.D., Andriantsitohaina R., Antoniou A., Arab T., Archer F., Atkin-Smith G.K. (2018). Minimal information for studies of extracellular vesicles 2018 (MISEV2018): A position statement of the International Society for Extracellular Vesicles and update of the MISEV2014 guidelines. J. Extracell. Vesicles.

[37] Lötvall J., Hill A.F., Hochberg F., Buzás E.I., Di Vizio D., Gardiner C., Gho Y.S., Kurochkin I.V., Mathivanan S., Quesenberry P. (2014). Minimal experimental requirements for definition of extracellular vesicles and their functions: A position statement from the International Society for Extracellular Vesicles. J. Extracell. Vesicles.

[38] Witwer K.W., Buzás E.I., Bemis L.T., Bora A., Lässer C., Lötvall J., Nolte-’t Hoen E.N., Piper M.G., Sivaraman S., Skog J. (2013). Standardization of sample collection, isolation and analysis methods in extracellular vesicle research. J. Extracell. Vesicles.

[39] Soekmadji C., Hill A.F., Wauben M.H., Buzás E.I., Di Vizio D., Gardiner C., Lötvall J., Sahoo S., Witwer K.W. (2018). Towards mechanisms and standardization in extracellular vesicle and extracellular RNA studies: Results of a worldwide survey. J. Extracell. Vesicles.

[40] Pegtel D.M., Gould S.J. (2019). Exosomes: Annual Review of Biochemistry. Annu. Rev. Biochem..

[41] Laulagnier K., Motta C., Hamdi S., Roy S., Fauvelle F., Pageaux J.-F., Kobayashi T., Salles J.-P., Perret B., Bonnerot C. (2004). Mast cell- and dendritic cell-derived exosomes display a specific lipid composition and an unusual membrane organization. Biochem. J..

[42] Qin B., Zhang Q., Hu X.M., Mi T.Y., Yu H.Y., Liu S.S., Zhang B., Tang M., Huang J.F., Xiong K. (2020). How Does Temperature Play a Role in the Storage of Extracellular Vesicles?. J. Cell. Physiol..

[43] Llorente A., Skotland T., Sylvänne T., Kauhanen D., Róg T., Orłowski A., Vattulainen I., Ekroos K., Sandvig K. (2013). Molecular lipidomics of exosomes released by PC-3 prostate cancer cells. Biochim. Biophys. Acta (BBA)-Mol. Cell Biol. Lipids.

[44] Yoshioka Y., Konishi Y., Kosaka N., Katsuda T., Kato T., Ochiya T. (2013). Comparative marker analysis of extracellular vesicles in different human cancer types. J. Extracell. Vesicles.

[45] Raposo G., Stoorvogel W. (2013). Extracellular vesicles: Exosomes, microvesicles, and friends. J. Cell Biol..

[46] Van Niel G., Porto-Carreiro I., Simoes S., Raposo G. (2006). Exosomes: A Common Pathway for a Specialized Function. J. Biochem..

[47] King H.W., Michael M.Z., Gleadle J.M. (2012). Hypoxic enhancement of exosome release by breast cancer cells. BMC Cancer.

[48] Keller S., König A.-K., Marmé F., Runz S., Wolterink S., Koensgen D., Mustea A., Sehouli J., Altevogt P. (2009). Systemic presence and tumor-growth promoting effect of ovarian carcinoma released exosomes. Cancer Lett..

[49] Kwok Z.H., Ni K., Jin Y. (2021). Extracellular Vesicle Associated Non-Coding RNAs in Lung Infections and Injury. Cells.

[50] Valadi H., Ekström K., Bossios A., Sjöstrand M., Lee J.J., Lötvall J.O. (2007). Exosome-mediated transfer of mRNAs and microRNAs is a novel mechanism of genetic exchange between cells. Nat. Cell Biol..

[51] Chang X., Fang L., Bai J., Wang Z. (2020). Characteristics and Changes of DNA in Extracellular Vesicles. DNA Cell Biol..

[52] Bellingham S.A., Guo B.B., Coleman B.M., Hill A.F. (2012). Exosomes: Vehicles for the Transfer of Toxic Proteins Associated with Neurodegenerative Diseases?. Front. Physiol..

[53] Feng D., Zhao W.-L., Ye Y.-Y., Bai X.-C., Liu R.-Q., Chang L.-F., Zhou Q., Sui S.-F. (2010). Cellular Internalization of Exosomes Occurs through Phagocytosis. Traffic.

[54] Jeyaram A., Jay S.M. (2018). Preservation and Storage Stability of Extracellular Vesicles for Therapeutic Applications. AAPS J..

[55] Maroto R., Zhao Y., Jamaluddin M., Popov V.L., Wang H., Kalubowilage M., Zhang Y., Luisi J., Sun H., Culbertson C.T. (2017). Effects of storage temperature on airway exosome integrity for diagnostic and functional analyses. J. Extracell. Vesicles.

[56] Oosthuyzen W., Sime N.E.L., Ivy J.R., Turtle E.J., Street J.M., Pound J., Bath L.E., Webb D.J., Gregory C.D., Bailey M.A. (2013). Quantification of human urinary exosomes by nanoparticle tracking analysis. J. Physiol..

[57] Madison M.N., Jones P.H., Okeoma C.M. (2015). Exosomes in human semen restrict HIV-1 transmission by vaginal cells and block intravaginal replication of LP-BM5 murine AIDS virus complex. Virology.

[58] Welch J.L., Madison M.N., Margolick J.B., Galvin S., Gupta P., Martínez-Maza O., Dash C., Okeoma C.M. (2017). Effect of prolonged freezing of semen on exosome recovery and biologic activity. Sci. Rep..

[59] Yuana Y., Böing A.N., Grootemaat A.E., van der Pol E., Hau C.M., Cizmar P., Buhr E., Sturk A., Nieuwland R. (2015). Handling and storage of human body fluids for analysis of extracellular vesicles. J. Extracell. Vesicles.

[60] Zhou H., Yuen P.S.T., Pisitkun T., Gonzales P.A., Yasuda H., Dear J.W., Gross P., Knepper M.A., Star R.A. (2006). Collection, storage, preservation, and normalization of human urinary exosomes for biomarker discovery. Kidney Int..

[61] Cheruvanky A., Zhou H., Pisitkun T., Kopp J.B., Knepper M.A., Yuen P.S.T., Star R.A. (2007). Rapid isolation of urinary exosomal biomarkers using a nanomembrane ultrafiltration concentrator. Am. J. Physiol. Ren. Physiol..

[62] Munagala R., Aqil F., Jeyabalan J., Gupta R.C. (2015). Bovine milk-derived exosomes for drug delivery. Cancer Lett..

[63] Agrawal A.K., Aqil F., Jeyabalan J., Spencer W.A., Beck J., Gachuki B.W., Alhakeem S.S., Oben K., Munagala R., Bondada S. (2017). Milk-derived exosomes for oral delivery of paclitaxel. Nanomed. Nanotechnol. Biol. Med..

[64] Zonneveld M.I., Brisson A.R., van Herwijnen M.J.C., Tan S., van de Lest C.H.A., Redegeld F.A., Garssen J., Wauben M.H.M., Nolte-’t Hoen E.N. (2014). Recovery of extracellular vesicles from human breast milk is influenced by sample collection and vesicle isolation procedures. J. Extracell. Vesicles.

[65] Jin Y., Chen K., Wang Z., Wang Y., Liu J., Lin L., Shao Y., Gao L., Yin H., Cui C. (2016). DNA in serum extracellular vesicles is stable under different storage conditions. BMC Cancer.

[66] Lacroix R., Judicone C., Poncelet P., Robert S., Arnaud L., Sampol J., Dignat-George F. (2012). Impact of pre-analytical parameters on the measurement of circulating microparticles: Towards standardization of protocol. J. Thromb. Haemost..

[67] Bæk R., Søndergaard E.K.L., Varming K., Jørgensen M.M. (2016). The impact of various preanalytical treatments on the phenotype of small extracellular vesicles in blood analyzed by protein microarray. J. Immunol. Methods.

[68] van Ierssel S.H., van Craenenbroeck E.M., Conraads V.M., van Tendeloo V.F., Vrints C.J., Jorens P.G., Hoymans V.Y. (2010). Flow cytometric detection of endothelial microparticles (EMP): Effects of centrifugation and storage alter with the phenotype studied. Thromb. Res..

[69] Tessier S.N., Bookstaver L.D., Angpraseuth C., Stannard C.J., Marques B., Ho U.K., Muzikansky A., Aldikacti B., Reátegui E., Rabe D.C. (2021). Isolation of intact extracellular vesicles from cryopreserved samples. PLoS ONE.

[70] Schubert P., Johnson L., Culibrk B., Chen Z., Tan S., Marks D.C., Devine D.V. (2021). Reconstituted cryopreserved platelets synthesize proteins during short-term storage and packaging a defined subset into microvesicles. Transfusion.

[71] Rubin O., Crettaz D., Canellini G., Tissot J.-D., Lion N. (2008). Microparticles in stored red blood cells: An approach using flow cytometry and proteomic tools. Vox Sang..

[72] Gelibter S., Marostica G., Mandelli A., Siciliani S., Podini P., Finardi A., Furlan R. (2022). The impact of storage on extracellular vesicles: A systematic study. J. Extracell. Vesicles.

[73] Sokolova V., Ludwig A.-K., Hornung S., Rotan O., Horn P.A., Epple M., Giebel B. (2011). Characterisation of exosomes derived from human cells by nanoparticle tracking analysis and scanning electron microscopy. Colloids Surf. B Biointerfaces.

[74] Lőrincz A.M., Timar C.I., Marosvári K.A., Veres D.S., Otrokocsi L., Kittel A., Ligeti E. (2014). Effect of storage on physical and functional properties of extracellular vesicles derived from neutrophilic granulocytes. J. Extracell. Vesicles.

[75] Deville S., Berckmans P., Van Hoof R., Lambrichts I., Salvati A., Nelissen I. (2021). Comparison of extracellular vesicle isolation and storage methods using high-sensitivity flow cytometry. PLoS ONE.

[76] Kumeda N., Ogawa Y., Akimoto Y., Kawakami H., Tsujimoto M., Yanoshita R. (2017). Characterization of Membrane Integrity and Morphological Stability of Human Salivary Exosomes. Biol. Pharm. Bull..

[77] Lee M., Ban J.-J., Im W., Kim M. (2016). Influence of storage condition on exosome recovery. Biotechnol. Bioprocess Eng..

[78] Wu Q., Zhou L., Lv D., Zhu X., Tang H. (2019). Exosome-mediated communication in the tumor microenvironment contributes to hepatocellular carcinoma development and progression. J. Hematol. Oncol..

[79] Park S.J., Jeon H., Yoo S.-M., Lee M.-S. (2018). The effect of storage temperature on the biological activity of extracellular vesicles for the complement system. Vitr. Cell. Dev. Biol.-Anim..

[80] Richter M., Fuhrmann K., Fuhrmann G. (2019). Evaluation of the Storage Stability of Extracellular Vesicles. J. Vis. Exp..

[81] Cheng Y., Zeng Q., Han Q., Xia W. (2018). Effect of pH, temperature and freezing-thawing on quantity changes and cellular uptake of exosomes. Protein Cell.

[82] Köberle V., Pleli T., Schmithals C., Augusto Alonso E., Haupenthal J., Bönig H., Peveling-Oberhag J., Biondi R.M., Zeuzem S., Kronenberger B. (2013). Differential Stability of Cell-Free Circulating microRNAs: Implications for Their Utilization as Biomarkers. PLoS ONE.

[83] Taylor D.D., Gercel-Taylor C. (2008). MicroRNA signatures of tumor-derived exosomes as diagnostic biomarkers of ovarian cancer. Gynecol. Oncol..

[84] Enderle D., Spiel A., Coticchia C.M., Berghoff E., Mueller R., Schlumpberger M., Sprenger-Haussels M., Shaffer J.M., Lader E., Skog J. (2015). Characterization of RNA from Exosomes and Other Extracellular Vesicles Isolated by a Novel Spin Column-Based Method. PLoS ONE.

[85] Ge Q., Zhou Y., Lu J., Bai Y., Xie X., Lu Z. (2014). miRNA in Plasma Exosome is Stable under Different Storage Conditions. Molecules.

[86] Baddela V.S., Nayan V., Rani P., Onteru S.K., Singh D. (2016). Physicochemical Biomolecular Insights into Buffalo Milk-Derived Nanovesicles. Appl. Biochem. Biotechnol..

[87] Sarker S., Scholz-Romero K., Perez A., Illanes S.E., Mitchell M.D., Rice G.E., Salomon C. (2014). Placenta-derived exosomes continuously increase in maternal circulation over the first trimester of pregnancy. J. Transl. Med..

[88] Ayers L., Kohler M., Harrison P., Sargent I., Dragovic R., Schaap M., Nieuwland R., Brooks S.A., Ferry B. (2011). Measurement of circulating cell-derived microparticles by flow cytometry: Sources of variability within the assay. Thromb. Res..

[89] Muller L., Hong C.-S., Stolz D.B., Watkins S.C., Whiteside T.L. (2014). Isolation of biologically-active exosomes from human plasma. J. Immunol. Methods.

[90] Nakase I., Ueno N., Matsuzawa M., Noguchi K., Hirano M., Omura M., Takenaka T., Sugiyama A., Kobayashi N.B., Hashimoto T. (2021). Environmental pH stress influences cellular secretion and uptake of extracellular vesicles. FEBS Open Bio.

[91] Qi H., Liu C., Long L., Ren Y., Zhang S., Chang X., Qian X., Jia H., Zhao J., Sun J. (2016). Blood Exosomes Endowed with Magnetic and Targeting Properties for Cancer Therapy. ACS Nano.

[92] Suharta S., Barlian A., Hidajah A.C., Notobroto H.B., Ana I.D., Indariani S., Wungu T.D.K., Wijaya C.H. (2021). Plant-Derived Exosome-like Nanoparticles: A Concise Review on Its Extraction Methods, Content, Bioactivities, and Potential as Functional Food Ingredient. J. Food Sci..

[93] Bahr M.M., Amer M.S., Abo-El-Sooud K., Abdallah A.N., El-Tookhy O.S. (2020). Preservation techniques of stem cells extracellular vesicles: A gate for manufacturing of clinical grade therapeutic extracellular vesicles and long-term clinical trials. Int. J. Vet. Sci. Med..

[94] Budgude P., Kale V., Vaidya A. (2021). Cryopreservation of mesenchymal stromal cell-derived extracellular vesicles using trehalose maintains their ability to expand hematopoietic stem cells in vitro. Cryobiology.

[95] Tegegn T.Z., De Paoli S.H., Orecna M., Elhelu O.K., Woodle S.A., Tarandovskiy I.D., Ovanesov M.V., Simak J. (2016). Characterization of procoagulant extracellular vesicles and platelet membrane disintegration in DMSO-cryopreserved platelets. J. Extracell. Vesicles.

[96] Zhang Y., Bi J., Huang J., Tang Y., Du S., Li P. (2020). Exosome: A Review of Its Classification, Isolation Techniques, Storage, Diagnostic and Targeted Therapy Applications. Int. J. Nanomed..

[97] Kreke M., Smith R., Hanscome P., Peck K., Ibrahim A. (2016). Processes for Producing Stable Exosome Formulations. U.S. Patent.

[98] Charoenviriyakul C., Takahashi Y., Nishikawa M., Takakura Y. (2018). Preservation of exosomes at room temperature using lyophilization. Int. J. Pharm..

[99] Akers J.C., Ramakrishnan V., Yang I., Hua W., Mao Y., Carter B.S., Chen C.C. (2016). Optimizing preservation of extracellular vesicular miRNAs derived from clinical cerebrospinal fluid. Cancer Biomark..

[100] Frank J., Richter M., De Rossi C., Lehr C.-M., Fuhrmann K., Fuhrmann G. (2018). Extracellular vesicles protect glucuronidase model enzymes during freeze-drying. Sci. Rep..

[101] Noguchi K., Hirano M., Hashimoto T., Yuba E., Takatani-Nakase T., Nakase I. (2019). Effects of Lyophilization of Arginine-rich Cell-penetrating Peptide-modified Extracellular Vesicles on Intracellular Delivery. Anticancer Res..

[102] El Baradie K.B.Y., Nouh M., O’Brien F., Liu Y., Fulzele S., Eroglu A., Hamrick M.W. (2020). Freeze-Dried Extracellular Vesicles From Adipose-Derived Stem Cells Prevent Hypoxia-Induced Muscle Cell Injury. Front. Cell Dev. Biol..

[103] Kusuma G.D., Barabadi M., Tan J.L., Morton D.A.V., Frith J.E., Lim R. (2018). To Protect and to Preserve: Novel Preservation Strategies for Extracellular Vesicles. Front. Pharmacol..

[104] Evtushenko E.G., Bagrov D.V., Lazarev V.N., Livshits M.A., Khomyakova E. (2020). Adsorption of extracellular vesicles onto the tube walls during storage in solution. PLoS ONE.

[105] van de Wakker S.I., van Oudheusden J., Mol E.A., Roefs M.T., Zheng W., Görgens A., El Andaloussi S., Sluijter J.P.G., Vader P. (2022). Influence of short term storage conditions, concentration methods and excipients on extracellular vesicle recovery and function. Eur. J. Pharm. Biopharm..

[106] Hermida-Nogueira L., Barrachina M.N., Izquierdo I., García-Vence M., Lacerenza S., Bravo S., Castrillo A., García Á. (2020). Proteomic analysis of extracellular vesicles derived from platelet concentrates treated with Mirasol® identifies biomarkers of platelet storage lesion. J. Proteom..

[107] Ashwood-Smith M.J. (1987). Mechanisms of cryoprotectant action. Symp. Soc. Exp. Biol..

[108] Bhattacharya S. (2018). Cryopretectants and Their Usage in Cryopreservation Process. Cryopreservation Biotechnology in Biomedical and Biological Sciences.

[109] Buchanan S.S., Gross S.A., Acker J.P., Toner M., Carpenter J.F., Pyatt D.W. (2004). Cryopreservation of Stem Cells Using Trehalose: Evaluation of the Method Using a Human Hematopoietic Cell Line. Stem Cells Dev..

[110] Eroglu A., Russo M.J., Bieganski R., Fowler A., Cheley S., Bayley H., Toner M. (2000). Intracellular trehalose improves the survival of cryopreserved mammalian cells. Nat. Biotechnol..

[111] Bari E., Perteghella S., Catenacci L., Sorlini M., Croce S., Mantelli M., Avanzini M.A., Sorrenti M., Torre M.L. (2019). Freeze-dried and GMP-compliant pharmaceuticals containing exosomes for acellular mesenchymal stromal cell immunomodulant therapy. Nanomedicine.

[112] Yong K.W., Safwani W.K.Z.W., Xu F., Abas W.A.B.W., Choi J.R., Pingguan-Murphy B. (2015). Cryopreservation of Human Mesenchymal Stem Cells for Clinical Applications: Current Methods and Challenges. Biopreserv. Biobank..

[113] Mandawala A.A., Harvey S.C., Roy T.K., Fowler K.E. (2016). Cryopreservation of animal oocytes and embryos: Current progress and future prospects. Theriogenology.

